# Adenoid ameloblastoma: Conservative approach in a rare odontogenic lesion

**DOI:** 10.1007/s10006-026-01547-3

**Published:** 2026-03-23

**Authors:** Eduardo dos Santos Vidal, William Cezar da Silveira, Camila Cerantula Moura, Hélen Kaline Farias Bezerra, Pablo Agustin Vargas, Juliana Lucena Schussel, Leandro Eduardo Klüppel, Heliton Gustavo de Lima

**Affiliations:** 1 Departament of Stomatology, Federal University of Paraná, Curitiba, Paraná Brazil; 2 Departament of Oral Diagnosis, Piracicaba Dental School, University of Campinas, Piracicaba, São Paulo, Brazil; 3 Departament of Anatomy, Federal University of Paraná, Curitiba, Paraná Brazil; 4 Departamento de Estomatologia, Universidade Federal do Paraná, Avenida Prefeito Lothário Meissner, 632, Jardim Botânico, Curitiba, CEP: 80210- 170 PR Brazil

**Keywords:** Ameloblastoma, Adenoid, Dentinoid, Odontogenic tumors, Head and Neck Neoplasms, Cryotherapy

## Abstract

**Background:**

Adenoid ameloblastoma (AA) is a newly recognized, rare odontogenic tumor variant, distinguished by histopathological features that combine characteristics of both ameloblastoma and adenomatoid odontogenic tumor.

**Case report:**

A 32-year-old female with an unilocular radiolucent lesion in the mandible associated with the impacted third molar, diagnosed as AA. Surgical management included complete lesion excision and cryotherapy. Despite the absence of significant symptoms, clinical surveillance is essential, as AA may have high recurrence rates. The recent inclusion of this entity in the WHO Head and Neck Tumor Classification (2022) highlights the need for accurate diagnosis and appropriate interventions to optimize patient prognosis.

**Conclusion:**

This report not only contributes to the growing understanding of this tumor biological behavior but also highlights the clinical relevance of documenting such cases to guide best practices and improve prognostic insights in rare odontogenic lesions.

**Supplementary Information:**

The online version contains supplementary material available at 10.1007/s10006-026-01547-3.

## Introduction

The adenoid ameloblastoma (AA) with dentinoid was first described in 1994 by Brannon, highlighting histopathological features that may include follicular or plexiform patterns with tubular structures similar to those found in adenomatoid odontogenic tumors (AOT) [1]. In 2004, Evans et al. further characterized AA as a tumor blending ameloblastoma and AOT features, including the formation of dentinoid tissue [2]. Since then, various terms, such as hybrid ameloblastoma and atypical AA, have been proposed for lesions exhibiting these unique patterns [3].

The WHO Classification of Head and Neck Tumors, updated in 2022, now formally recognizes AA as a distinct entity with essential and desirable diagnostic features, including its cribriform architecture, adenoid structures, and dentinoid formation. While duct-like or glandular structures are rarely observed in ameloblastomas, these characteristics support the adenoid subtype as a unique diagnostic entity, despite the challenges it presents [2, 4–8]. In this article, we present a new case of AA and discuss a conservative surgical approach for managing this recent, intriguing, and rare lesion.

## Case Report

**Fig. 1:**
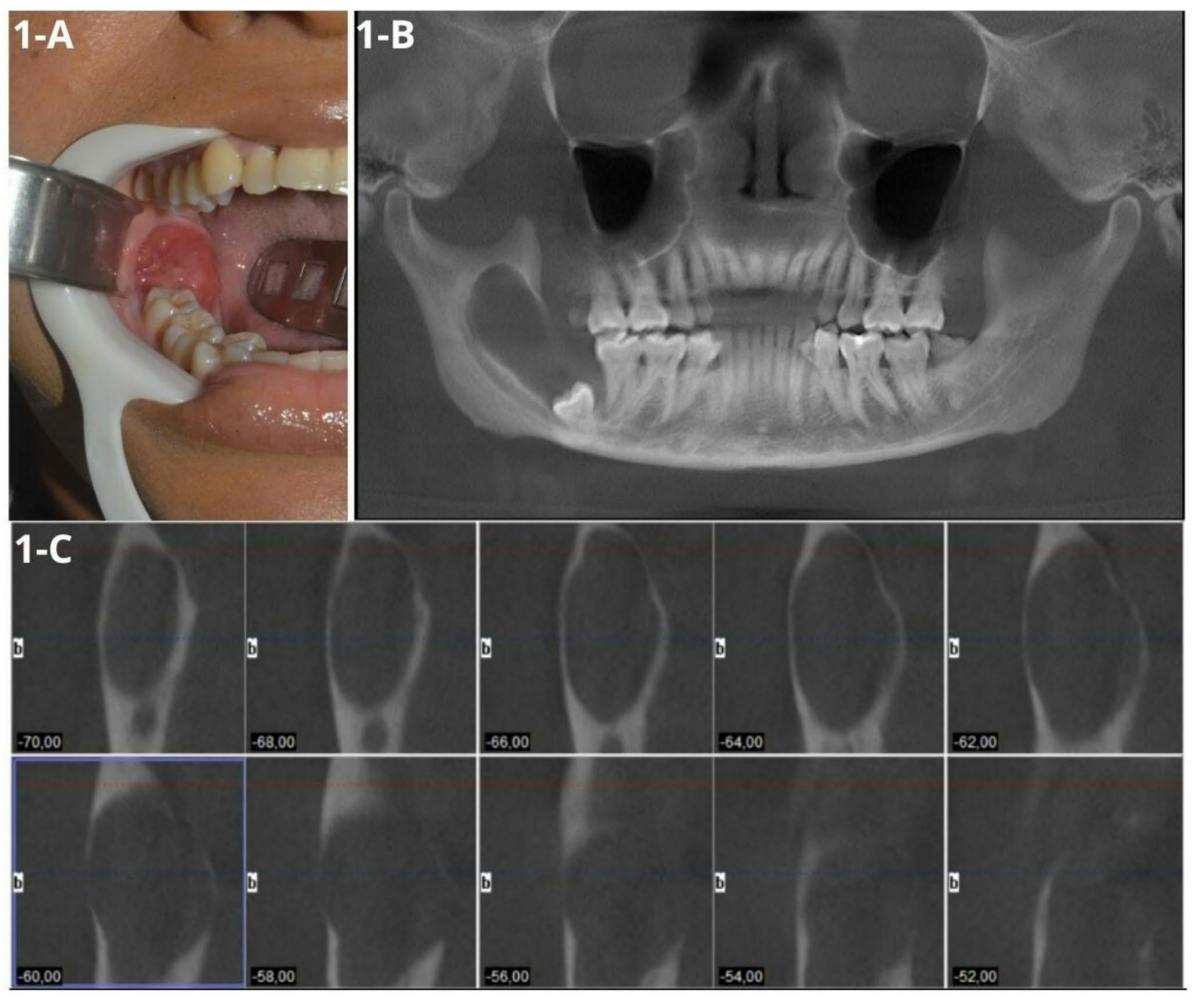
Clinical and tomographic features. **(A)** Intraoral view showing a sessile nodule within the oral mucosa in the posterior region of the right mandible. **(B)** Panoramic reconstruction revealing a corticated unilocular hypodense osteolytic lesion with displacement of the lower right third molar. **(C)** Parasagittal reconstruction of the right mandibular side, demonstrating fenestration in the lingual cortical bone

A 32-year-old female patient was referred to the Oral and Maxillofacial Surgery and Traumatology Service at the Federal University of Paraná (UFPR). Her medical history included hypothyroidism and obesity, and there were no apparent abnormalities on general physical evaluation. Intraoral examination revealed an extensive lobulated mass located in the posterior region of the right mandible, approximately 2 cm in size, erythematous in color, with a smooth surface, regular contour, firm on palpation, and with a history of slow growth over approximately three years, asymptomatic (Fig. 1A). Panoramic radiography showed an extensive radiolucent lesion involving the right mandibular body and ramus, associated with the lower third molar. Cone beam computed tomography (CBCT) was requested, revealing an expansive, hypodense, unilocular lesion with well-defined borders, extending from the mandibular body and ramus to the mandibular notch, with inferior displacement of the lower third molar and adjacent bone resorption near the lower second molar (Fig. 1B). Parassagittal sections showed bone fenestration, mainly in the lingual cortical plate, and proximity of the lesion to the inferior alveolar nerve (Fig. 1C) .

**Fig. 2:**
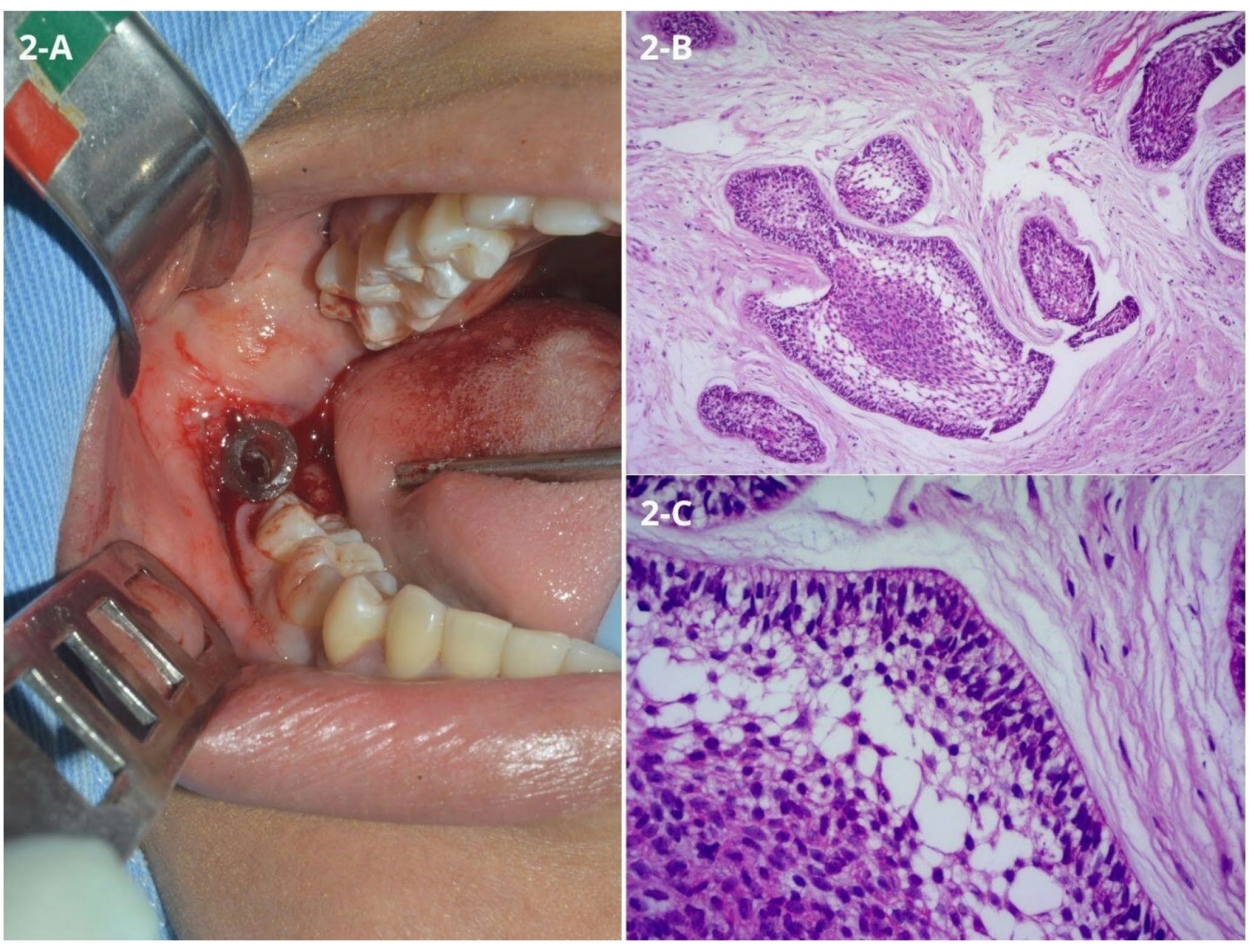
Initial approach and histopathological features. **(A)** Drain placement after incisional biopsy. **(B)** Fibrous connective tissue containing ameloblastomatous islands with squamous metaplasia, resulting in acanthomatous pattern – Hematoxylin and Eosin (HE) 100x. **(C)** Islands with peripheral cells showing inverted nuclear polarity, vacuolization of the cytoplasm, referring to pre-ameloblasts. Adjacent, stellate reticulum-like cells and others exhibiting squamous differentiation - HE 400x

Based on the clinical and radiographic findings, the differential diagnoses included dentigerous cyst, unicystic ameloblastoma, and odontogenic keratocyst. Initial treatment involved performing an incisional biopsy and placing a drain for decompression during the same surgical procedure (Fig. 2A). Histopathological analysis revealed fibrous connective tissue containing islands of ameloblast-like cells with peripheral columnar basal cells arranged in palisades, showing cytoplasmic vacuolization, hyperchromatic, and polarized nuclei. Adjacent to these peripheral cells, areas resembling the stellate reticulum of the enamel organ were observed. Some central cells within the islands exhibited squamous metaplasia, supporting the diagnosis of acanthomatous ameloblastoma (Fig. 2B-C).

**Fig. 3:**
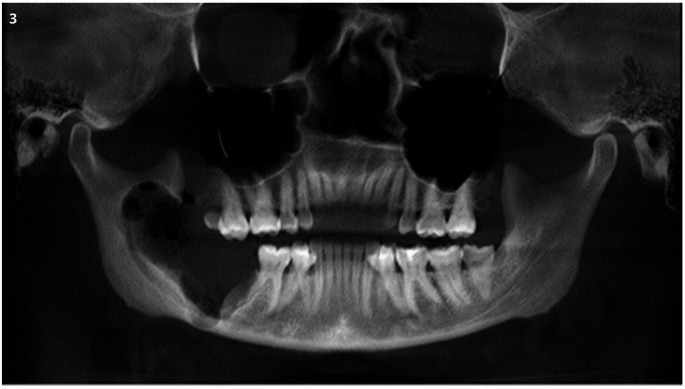
CBCT. Panoramic reconstruction showing the extension of surgical site in immediate pos-operatory

Following the initial procedure, the patient did not attend scheduled follow-ups and returned for reassessment seven months later, reporting that the drain had detached after one month. A new CBCT scan indicated a slight increase in lesion size. The proposed treatment involved surgical enucleation under general anesthesia, including the extraction of teeth 47 and 48, followed by adjuvant cryotherapy, which involved the application of liquid nitrogen to the bony cavity and its margins in five one-minute sessions. This approach aimed to induce thermal necrosis of any remaining neoplastic cells. The procedure was completed successfully without complications (Fig. 3).

**Fig. 4:**
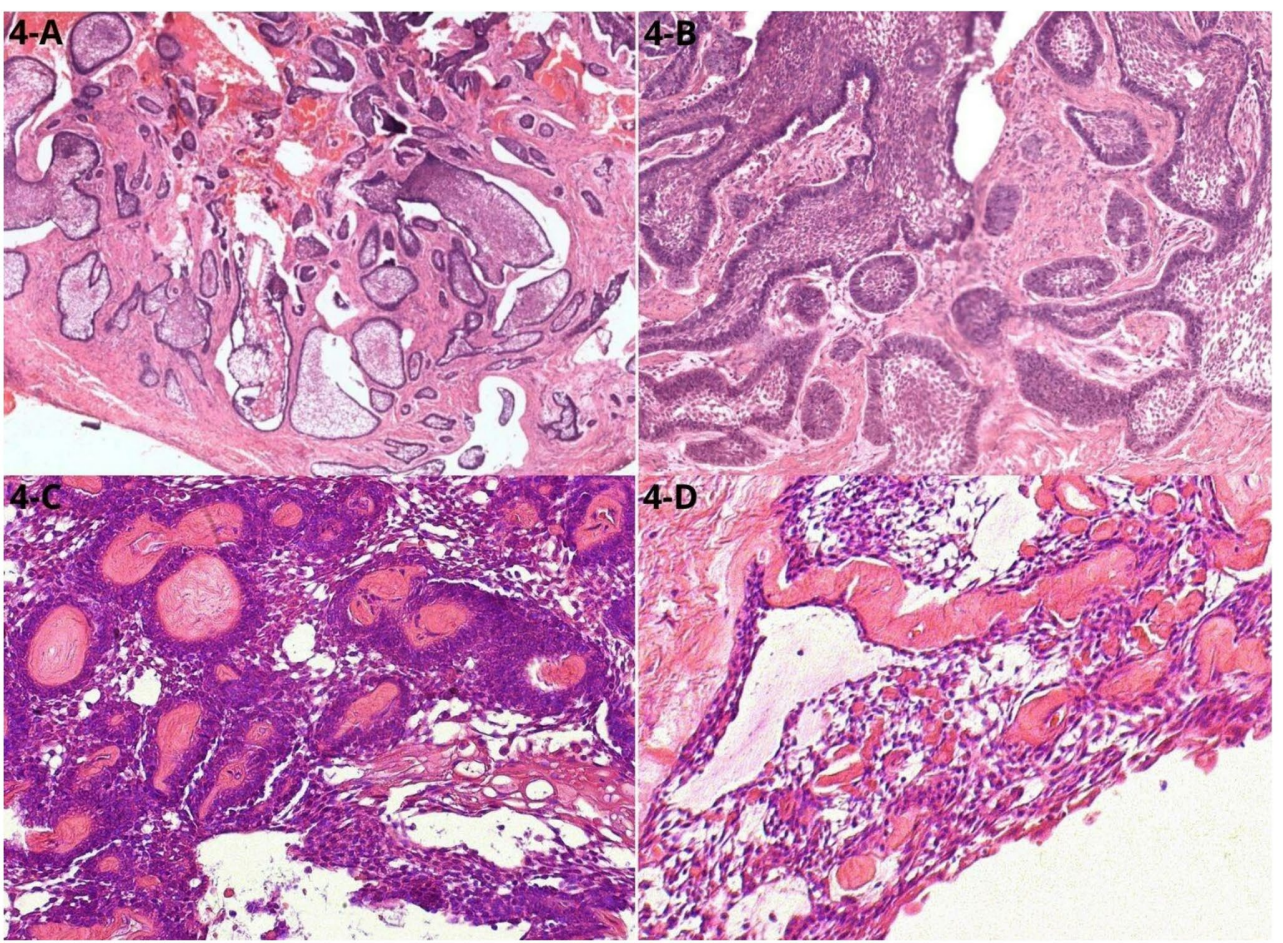
Histopathological aspects of the excisional specimen. **(A)** Numerous ameloblastomatous islands, varying in size HE 40x. **(B)** Magnification of microscopy HE 200x. **(C)** Islands with hyperchromatic cells, around dentinoid material, organized, in some regions, in a cribriform shape, similar to ducts. **(C)** Area of cells like stellate reticulum associated with dentinoid material

Microscopic examination confirmed the presence of ameloblastoma with follicular and plexiform patterns, cystic microcavities, and areas exhibiting duct-like morphology (Fig. 4A-C).

**Fig. 5:**
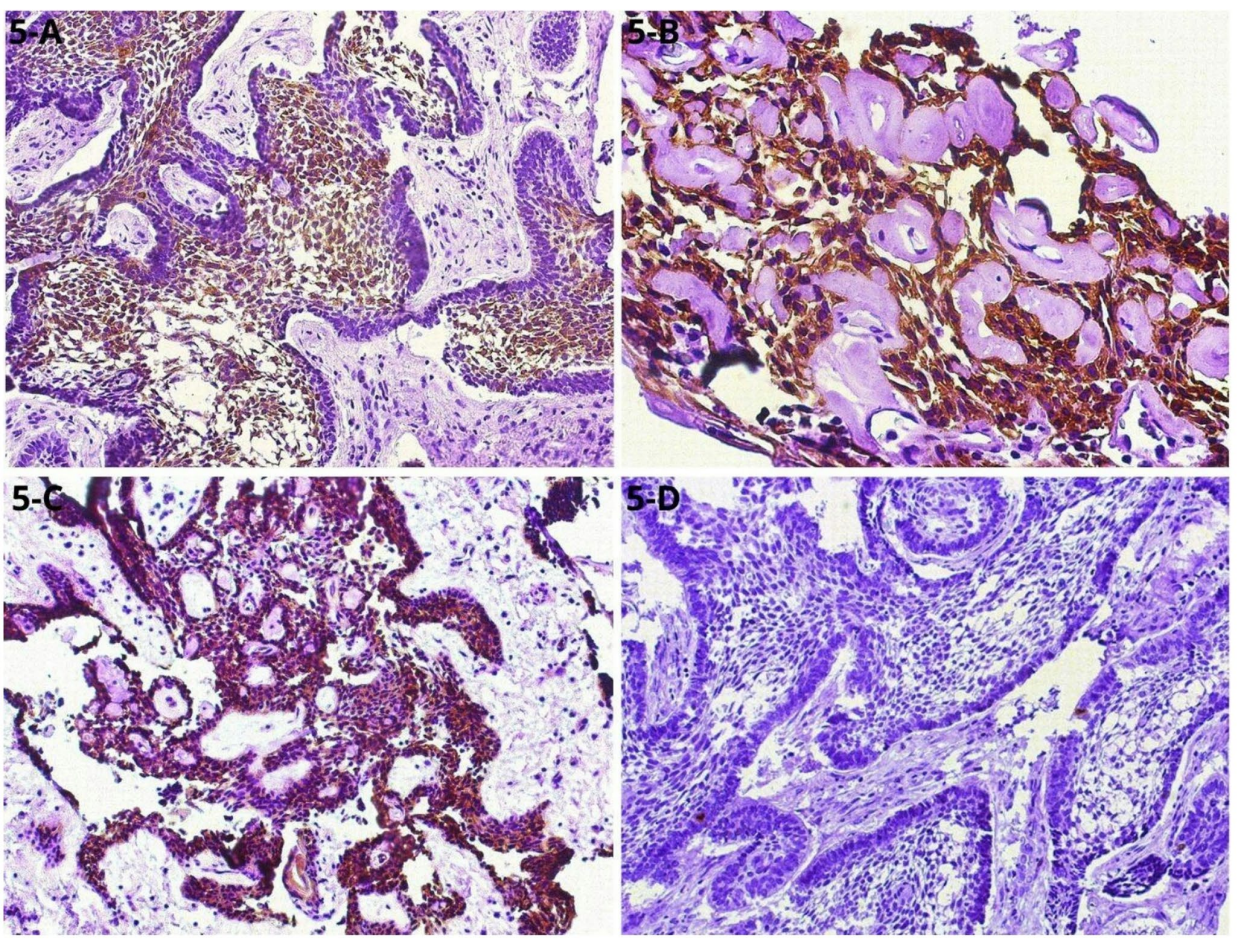
Immunohistochemical findings. **(A)** Tumor islands of ameloblastoma with positive staining for CK19 in the central stellate reticulum-like cells **(**200x); **(B)** CK14-positive cells associated with dentinoid material (400x); **(C)** β-Catenin-positive ameloblastoma tumor cells (100x); **(D)** Ki-67 showing the low proliferation rate (< 5%) (100x)

Eosinophilic, amorphous material resembling dentinoid was observed interspersed among the tumor’s epithelial cells. Additionally, positive immunohistochemical staining for CK19, CK14, and β-catenin, along with a low Ki-67 proliferation index (< 5%), supported the diagnosis (Fig. 5A-D).

**Fig. 6:**
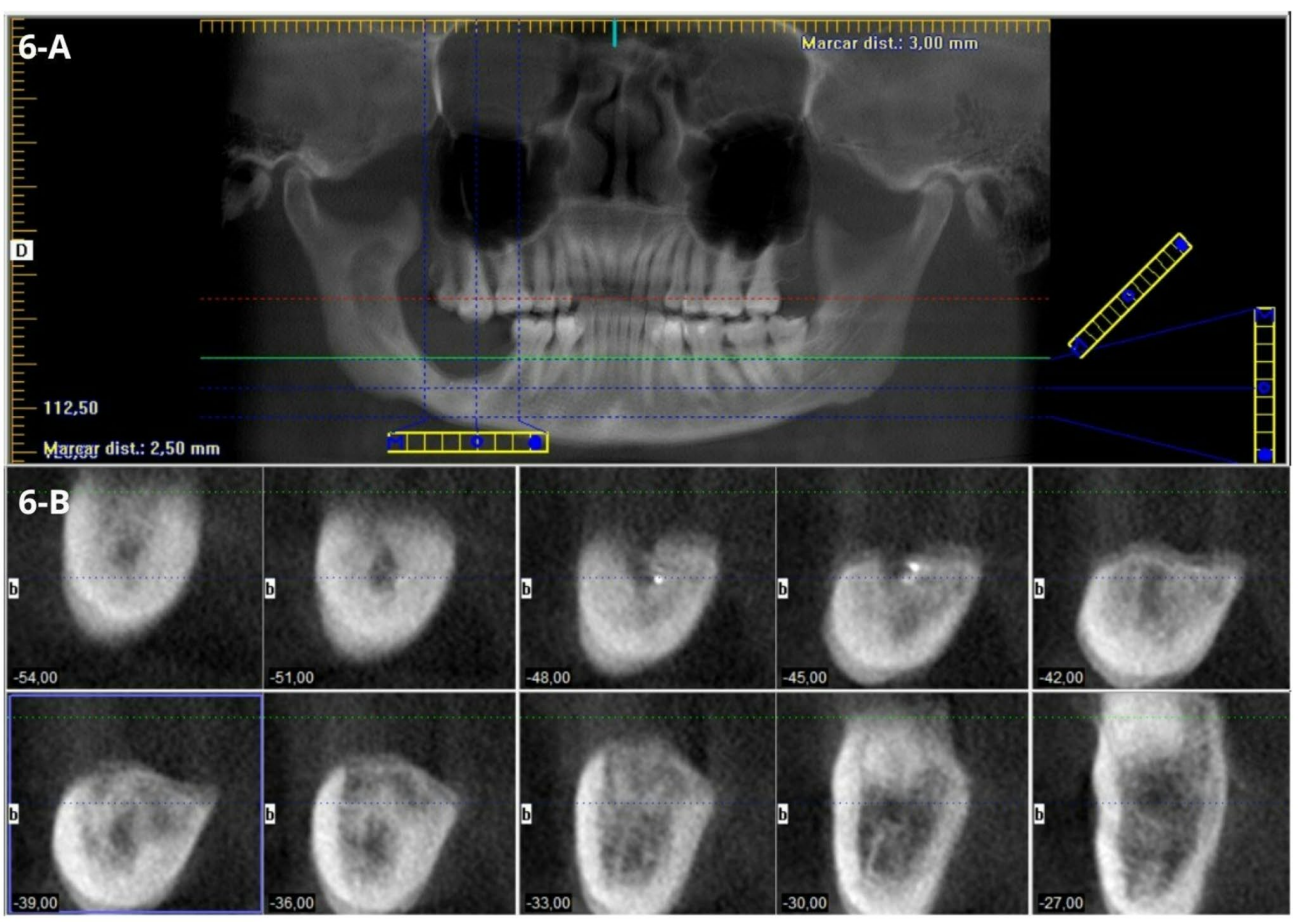
CBCT. **(A)** Panoramic reconstruction at 18 months of pos-operatory follow-up. **(B)** Parassagital reconstruction revealing bone repair and no signs of recurrence

These histopathological findings confirmed a revised diagnosis of Adenoid Ameloblastoma. During an 18-month follow-up period, no recurrence was noted, and CBCT scans indicated evidence of bone repair (Fig. 6A-B).

## Discussion

The identification of histopathological features that link ameloblastoma with AOT is of considerable importance, particularly in assessing the clinical behavior and prognosis of this rare odontogenic neoplasm. AA, recently included in the WHO Classification of Head and Neck Tumors (2022), presents both ameloblastoma and AOT-like features, such as duct-like structures and cribriform architecture, which are unusual in traditional ameloblastomas [3].The presence of dentinoid material further supports this diagnosis, as its occurrence in approximately 70% of the cases analyzed by Sachdev et al. [3] demonstrates that it represents a frequent and relevant characteristic, aligning with Brannon’s 1994 recognition of AA as a distinct entity [1]. 

In our reported case of a 32-year-old patient diagnosed with AA, we emphasize the importance of understanding this rare variant for accurate diagnosis and effective management. In this case the identified structures were a determining factor for the diagnosis, especially considering the similarity between adenoid ameloblastoma and adenomatoid odontogenic tumor. In this case, the lesion was initially diagnosed on incisional biopsy as conventional ameloblastoma, acanthomatous variant, and was later reclassified as a different entity after excisional biopsy. AA can present more complex clinical behaviors, necessitating personalized therapeutic approaches to optimize disease control. Literature suggests that the treatment of such lesions should be approached with caution to ensure complete tumor eradication while minimizing recurrence rates [9]. In this case, the surgical management included the extraction of involved teeth, complete excision of the lesion, and the use of cryotherapy, complemented by the application of vancomycin paste and a hemostatic agent. Cryotherapy has emerged as a promising adjunctive treatment for aggressive odontogenic lesions, effectively destroying residual tumor cells and thereby reducing the risk of recurrence [10]. Notably, to our knowledge, this is the first case documented utilizing a conservative approach that incorporates cryotherapy for the management of AA. In contrast to other well-established surgical approaches for conventional ameloblastoma, which typically suggest resection with an osseus safety margin.

The clinical approach such as this, despite demonstrating no signs of recurrence at 18 months post-operation, still necessitates follow-up for at least 5 years. This prolonged observation period is crucial for obtaining strong evidence of no further recurrences, as there is currently no consensus on the average duration required for follow-up principally for AA [9–12]. Loyola et al. (2015) further highlighted this need by reporting recurrences in all five of their cases, with intervals extending up to 282 months, and recurrence in 71% of cases in their systematic review, underscoring the importance of strict and prolonged surveillance [6]. 

There is still no consensus on the length of time a patient with AA should be followed up after surgical treatment. However, based on the articles we found, along with the information in this report, we have an average follow-up period of 41,1 months without recurrence, as shown in Table 1. This table also clarifies the recurrence of the lesion up to the last successful treatment, as well as its location and the treatment performed.

**Table 1:** – FOLLOW-UP OF ALL CASES WITH DIAGNOSYS OF AA

Author’s / Year	*N* of cases of AA	Case	Localization	Number of recurrences independent of treatment.	Last surgical treatment	Follow-up (months)
Slabbert et al. 1992 [11]	1	1	Mandibule	0	Wide Excision	N/D
Matsumoto et al. 2001 [12]	1	1	Mandibule	1	Wide Excision	30
Evans et al. 2004 [2]	1	1	Anterior Mandibule	1	Ressection	18
Zhang et al. 2006 [13]	1	1	Bilateral Mandibule	0	Ressection	36
Jivan et al. 2007 [14]	1	1	Bilateral Mandibule	N/D	N/D	N/D
Ghasemi-Moridani and Yazdi 2008 [15]	1	1	Maxilla	0	Excision	N/D
Ide et al. 2009 [16]	1	1	Bilateral Maxilla	3	Excision	96
Sonone et al. 2011 [17]	1	1	Mandibule	0	Ressection	6
Sexena et al. 2012 [18]	1	1	Maxilla	3	Partial Maxillectomy	N/D
Kumar et al. 2013 [19]	1	1	Mandibule	0	Ressection	36
Yamazaki et al. 2014 [20]	1	1	Mandibule	0	Ressection	36
Loyola et al. 2015 [6]	5	1	Posterior Mandibule	1	Ressection	108
		2	Posterior Maxilla	9	Ressection + Chemoterapy	Alive with dicease - Loss of follow-up
		3	Posterior Maxilla	5	Ressection + Radiotherapy	76
		4	Anterior Maxilla	5	Ressection	282
		5	Posterior Maxilla	2	Ressection + Radiotherapy	52
Salehinejad et al. 2016 [21]	1	1	Maxilla	9	Ressection	19
Khalele et al. 2016 [22]	1	1	Mandibule	0	Hemimandibulectomy	14
Rai et al. 2017 [5]	1	1	Mandibule	0	Enucleation	N/D
Sathyanarayan et al. 2017 [23]	1	1	Bilateral Maxilla	5	Ressection	76
Adorno-Farias et al. 2018 [4]	8	1	Mandibule	0	Ressection	N/D
		2	N/D	0	Ressection	N/D
		3	Mandibule	0	Ressection	N/D
		4	Posterior Mandibule	0	Ressection	N/D
		5	N/D	0	Ressection	N/D
		6	Anterior Mandibule	0	Ressection	N/D
		7	Posterior Mandibule	0	Ressection	N/D
		8	Mandibule	0	Ressection	N/D
Arruda et al. 2020 [24]	1	1	Maxilla	0	Ressection	36
Lathakumari et al. 2021 [25]	1	1	Posterior Maxilla	3	Excision + curettage	24
Jofre et al. 2022 [26]	1	1	Maxilla	N/D	Endoscopic Medial Maxilectomy	N/D
Jayasooriya et al. 2022 [27]	3	1	Anterior and Posterior Mandibule	1	Mandibulectomy	N/D
		2	Posterior Mandibule	3	N/D	N/D
		3	Anterior Maxilla	0	Excision	6
Ramani et al. 2023 [28]	1	1	Posterior Mandibule	0	Excision	2
Pandiar et al. [29]	1	1	Anterior Mandibule	0	Mandibulectomy	N/D
Silver et al. 2023 [30]	1	1	Maxilla	0	Partial Maxillectomy	N/D
Alramadhan et al. 2023 [31]	1	1	Posterior and Anterior Mandibule	0	Excision	6
Khalaj et al. 2023 [32]	4	1	Mandibule	0	Hemimandibulectomy	33
		2	Mandibule	0	Surgical Removal	16
		3	Mandibule	0	Surgical Removal	6
		4	Mandibule	1	Surgical Removal	25
Jabbar el al. 2023 [33]	2	1	Mandibule	0	Incomplete excision	N/D
		2	Maxilla	2	N/D	N/D
Chettiankandy et al. 2023 [34]	1	1	Mandibule	0	Excision	24
Sharma et al. 2023 [8]	2	1	Posterior Mandibule	N/D	Hemimandibulectomy	N/D
		2	Anterior Mandibule	N/D	N/D	N/D
Keshwar et al. 2024 [35]	1	1	Anterior Mandibule	N/D	Excision	Loss of follow-up
Yang et al. 2024 [36]	5	1	Posterior and Anterior Maxilla	N/D	Partial Maxillectomy	N/D
		2	Posterior and Anterior Maxilla	0	Partial Maxillectomy	14
		3	Posterior Mandibule	4	Marsupialization	4
		4	Posterior and Anterior Maxilla	0	Curettage	N/D
		5	Posterior Maxilla	0	Curettage	138
Torres et al. 2024 [37]	1	1	Anterior Mandibule	0	Enucleation + Carnoy’s Solution	25
Lin et al. 2025 [38]	1	1	Posterior Maxilla	0	Excision	48
Gangadharan 2025 [39]	1	1	Mandibule	0	Wide Excision	12
Datar et al. 2025 [40]	1	1	Posterior Mandibule	1	Hemimandibulectomy	18
Haraguchi et al. 2025 [41]	1	1	Posterior Mandibule	1	Ressection	32
Titinchi et al. 2025 [42]	1	1	Posterior and Anterior Mandibule	0	Ressection	24
Current case	1	1	Posterior Mandibule	0	Enucleation + Cryotherapy	18
**Total cases**	**60**		**% of 1 or more recurrences of AA**	**31**,**67**	**Average of months in follow-up without recurrence**	**41**,**1**

Table 1: Representing the follow-up time for diagnosed cases of AA, indication of regions of occurrences, recurrence of the lesion regardless of treatment and percentage of recurrences. N/D = No data

Histopathological examination showed ameloblastomatous islands with palisading cells and an acanthomatous pattern, alongside areas with cystic microcavities, stellate reticulum-like epithelium, cribriform architecture, and dentinoid material. These findings reflect a unique mixed morphological pattern consistent with lesions described in the literature, merging characteristics of ameloblastoma and AOT [4]. Jayasooriya et al. (2022) emphasized that lesions raising diagnostic doubt between AA and AOT should be carefully evaluated for the presence of infiltrative growth and an ameloblastomatous component. Although AA may exhibit duct-like structures, glandular differentiation, and epithelial whorls that closely resemble the rosette-like and ductiform arrangements seen in AOT, the architectural context differs significantly. In AOT, these structures are typically organized within a well-circumscribed lesion lacking an infiltrative ameloblastomatous pattern. In contrast, AA demonstrates areas of conventional ameloblastoma, including peripheral palisading, reverse nuclear polarization, and stromal invasion, features that are not characteristic of AOT [27]. Therefore, the coexistence of adenoid-like structures with a true ameloblastomatous component supports the diagnosis of AA rather than AOT, even when glandular or duct-like formations are prominent.

Morais et al. (2023) reported that 93.3% of AA cases exhibit odontogenic epithelium similar to stellate reticulum, with cribriform structures in 90% of cases and duct-like features in 100%, further corroborating the present findings [10]. Furthermore, Dentinogenic Ghost Cell Tumor (DGCT) can be considered as a differential diagnosis, owing to the presence of ghost cells, ameloblastoma-like epithelium, dentinoid material deposition, and the potential occurrence of duct-like structures and a cribriform architecture. Consequently, studies suggest that DGCT, similar to AA, may contain mutations in the *CTNNB1/WNT pathway* [36, 43, 44]. 

Recent molecular investigations have expanded the understanding of Adenoid Ameloblastoma (AA). Fonseca et al. (2026) reported recurrent alterations in the *CTNNB1* gene, suggesting activation of the Wnt/β-catenin signaling pathway as a potential driver event in a subset of cases. However, *CTNNB1* variants appear to be heterogeneous and are not identified in all reported samples, indicating molecular variability within this entity. Notably, mutations commonly observed in conventional ameloblastoma *(BRAF p.V600E)* and in adenomatoid odontogenic tumor *(KRAS)* are generally absent in AA, although isolated reports of *BRAF* positivity have been described. These findings suggest that AA may present a molecular profile that partially diverges from other odontogenic epithelial tumors, reinforcing its recognition as a distinct entity while also highlighting the need for further molecular studies [45]. 

The absence of symptoms such as pain or paresthesia in our patient contributed to a delayed diagnosis and subsequent early intervention. Literature indicates that swelling is the most common clinical presentation in AAs, occurring in 53.3% of cases, while pain and paresthesia are less frequent, at 13.3% and 10%, respectively [10]. This underscores the necessity for ongoing clinical and radiographic surveillance to facilitate early identification of asymptomatic odontogenic lesions and prevent disease progression.

Prognostically, AA shares a tendency for recurrence with conventional ameloblastoma, particularly if excision is incomplete. Recurrence rates range from 15% to 30%, even after adequate surgical treatment, with recurrence being especially common in cases involving conservative or incomplete resections [6]. Given that our case involved a unilocular lesion with well-defined borders, we opted for a conservative treatment approach, achieving complete resection of the lesion while employing adjunctive cryotherapy to further minimize recurrence risk. Nonetheless, long-term follow-up with regular clinical examinations and imaging studies remains essential to detect early signs of recurrence and ensure effective disease control.

This case report contributes significantly to the literature by documenting a rare lesion and providing detailed insights into its diagnosis, management, and follow-up. It reinforces the value of a multidisciplinary approach to treating odontogenic tumors and highlights the importance of integrating clinical, radiographic, and histopathological data to enhance our understanding of this neoplasm’s biology. Such comprehensive knowledge can facilitate the development of more precise interventions for future clinical management, ultimately improving patient outcomes [7].

## Conclusion

In summary, this case underscores the distinct histopathological features of AA and highlights conservative treatment with adjunctive cryotherapy as a promising approach to reduce recurrence risk while preserving tissue. Ongoing follow-up remains crucial to ensure effective management of this rare entity.

## Supplementary Information

Below is the link to the electronic supplementary material.

Supplementary Material 1

## Data Availability

No datasets were generated or analysed during the current study.
