# Correction to: Adenoid ameloblastoma: Conservative approach in a rare odontogenic lesion

**DOI:** 10.1007/s10006-026-01559-z

**Published:** 2026-04-18

**Authors:** Eduardo dos Santos Vidal, William Cezar da Silveira, Camila Cerantula Moura, Hélen Kaline Farias Bezerra, Pablo Agustin Vargas, Juliana Lucena Schussel, Leandro Eduardo Klüppel, Heliton Gustavo de Lima

**Affiliations:** 1 Departament of Stomatology, Federal University of Paraná, Curitiba, Paraná Brazil; 2 Departament of Oral Diagnosis, Piracicaba Dental School, University of Campinas, Piracicaba, São Paulo, Brazil; 3 Departament of Anatomy, Federal University of Paraná, Curitiba, Paraná Brazil; 4 Departamento de Estomatologia, Universidade Federal do Paraná, Avenida Prefeito Lothário Meissner, 632, Jardim Botânico, Curitiba, PR CEP: 80210- 170 Brazil

**Correction to: Oral and Maxillofacial Surgery**

10.1007/s10006-026-01547-3

In the original version of the manuscript, reference 30 was unintentionally removed instead of being corrected and updated, and it has now been reinstated.

The original article has been corrected.

