# Supplementary material for: Adenoid ameloblastoma: Conservative approach in a rare odontogenic lesion
Source: Oral Maxillofac Surg. 2026 Mar 23;30(1):61. doi: 10.1007/s10006-026-01547-3 (PMC13006472; doi:10.1007/s10006-026-01547-3)
Supplement: Supplementary file 1 — Supplementary Material 1 [file 10006_2026_1547_MOESM1_ESM.docx]

**Adenoid Ameloblastoma: Conservative Approach in a Rare Lesion**

Eduardo dos Santos Vidal^a^, William Cezar da Silveira^a^, Camila Cerantula Moura^a^, Hélen Kaline Farias Bezerra^b^, Pablo Agustin Vargas^b^, Juliana Lucena Schussel^a^, Leandro Eduardo Klüppel^c^, Heliton Gustavo de Lima^a^

**^a^** Departament of Stomatology, Federal University of Paraná, Curitiba, Paraná, Brazil.

**^b^** Departament of Oral Diagnosis, Piracicaba Dental School, University of Campinas, Piracicaba, São Paulo, Brazil.
**^c^** Departament of Anatomy, Federal University of Paraná, Curitiba, Paraná, Brazil.

Corresponding author:

Heliton Gustavo de Lima, Departamento de Estomatologia, Universidade Federal do Paraná. Avenida Prefeito Lothário Meissner, 632. CEP: 80210-170, Jardim Botânico, Curitiba, PR, Brazil.

Email address: [helitonlima@ufpr.br](mailto:helitonlima@ufpr.br)

**Declaration of competing interest**

The authors declare that they have no competing interest.
